# Measurement Model of Women’s Preferences in Obstetrician and Gynecologist Selection in the Private Sector: Exploratory and Confirmatory Factor Analysis

**DOI:** 10.30476/IJCBNM.2020.82278.1049

**Published:** 2020-04

**Authors:** Masood Setoodefar, Hamed Tabesh, Fatemeh Tara, Saeed Eslami, Fatemeh Heshmati Nabavi, Najmeh Valizadeh Zare, Seyyed Hassan Taheri, Mohammad Reza Rajabzadeh Moghaddam, Kobra Etminani

**Affiliations:** 1 Department of Medical Informatics, School of Medicine, Mashhad University of Medical Sciences, Mashhad, Iran; 2 Patient Safety Research Center, Mashhad University of Medical Sciences, Mashhad, Iran; 3 Nursing and Midwifery Care Research Center, Mashhad University of Medical Sciences, Mashhad, Iran; 4 Department of Computer Sciences, School of Engineering, Khayyam University, Mashhad, Iran; 5 Department of Pure Mathematics, Center of Excellence in Analysis on Algebraic Structures (CEAAS), Ferdowsi University, Mashhad, Iran

**Keywords:** Factor analysis, Obstetric and gynecologic patients, Patient preferences, Women’s health services

## Abstract

**Background:**

The purpose of this study is to construct and validate a measurement model of women’s preferences in Obstetrician and Gynecologist (OB/GYN) selection in the private sector of non-clinical parameters.

**Methods:**

This methodological study included 462 respondents in OB/GYN’s offices to a researcher-made questionnaire. The patients visited 57 offices of OB/GYNs in the city of Mashhad in Iran and completed women’s preferences in OB/GYN selection questionnaire over a 2-month period from January to February 2018. Exploratory Factor Analysis (EFA) was conducted to verify the instrument’s construct validity. Confirmatory Factor Analysis (CFA) was used to test whether the data fit our hypothesized model obtained from EFA model.

**Results:**

The first draft of the questionnaire was prepared with 118 items based on literature review. The outcome of content validity assessment was a 51-item questionnaire. Scale-Content Validity Index (S-CVI) turned out to be 0.80. The results of EFA yielded an instrument with 33 items in six domains, which explained 52.657% of the total variance of the questionnaire. With performing CFA, the 6-factor model with 29 items demonstrated a good fit with the data (CFI=0.952, CMIN/DF=1.613, RMSEA=0.036). Availability and Accessibility, Communicational Skills, Office Environment, Recommendation by Others, Special Services, and Cost and Insurance were found to define the women’s preferences in OB/GYN selection in private sector, Iran.

**Conclusion:**

The developed measurement model considers the patient’s preferences that influence decision-making process on OB/GYN selection. It can provide useful knowledge for OB/GYNs and policymakers to design appropriate and efficient marketing strategies according to the consumer preferences priority.

## INTRODUCTION

Choosing a doctor is a multi-dimensional process that involves the patient’s needs and preferences. ^1^ Although the clinical quality of physicians is an influential component in this process, ^2^ other non-clinical factors, including “respect to dignity,” “respect for autonomy,” “respect for confidentiality,” “proper communication,” “access to prompt attention,” “quality of basic amenities,” and “choice of care provider” directly affect the patient’s attitudes. ^3^

Pregnancy issues beside prevention and early detection of cervical cancer and breast cancer are some crucial practices that need routine health check-ups. Several studies explain problem of low reporting rates across routine health check-ups. Providing patient-centered care affects the patient’s communication and satisfaction. It can lead to better treatment and health outcomes. ^4^

The special conditions of OB/GYN’s medical examinations lead to women’s preferences and attitudes playing more critical role in OB/GYN’s selection than other physicians. ^5^ Previous researchers in this area have usually examined the impact of cultural factors, religion, social habits, and beliefs on the gender preference in OB/GYN selection. ^6 - 11^ Most of the studies based in Western societies have revealed that a physician’s specialty, experience, professionalism and clinical skills are the most important factors. ^12 , 13^ Other studies have found that behavioral and communication skills affect the patient’s satisfaction. ^4 , 14^ There are some shortcomings in previous studies, such as lack of validity and reliability in the questionnaires used, focusing on OB/GYN’s gender and ignoring other preference dimensions such as non-clinical factors. 

The aim of this study was to build a measurement model to examine the relationship between the latent variables and their measures in OB/GYN selection. Such a model can also demonstrate the simultaneous effect of these preferences on the women’s decision-making process. To this end, the research team developed a valid and reliable questionnaire to identify the non-clinical factors of women’s preferences when selecting an OB/GYN in the private sector.

## MATERIALS AND METHODS

This methodological study conducted on female patients in obstetrics and gynecology offices in Mashhad, Iran 2018. Mashhad with a population of 3 million is the second-largest Iranian city. It is recognized as the east headquarter of the country that delivers services to the patients from all over the province, as well as health tourists from neighboring countries. ^15^

The target sample size was determined as 500 participants, according to the number of items in the questionnaire (item: participant ratio; 1:10). In order to collect a representative sample from an economic, social, and cultural perspective, a proportionate stratified sample by region was conducted at the office level. The total number of active OB/GYN offices in the city is about 255, which are distributed in 10 regions. The research team randomly selected proportional number of offices from each region by choosing a sampling fraction 1/4. During a scheduled visit day in each office, all patients over 18 years old who provided verbal informed consent were eligible to participate in the study. Individuals were included only if they voluntarily agreed to participate. Patients in a hurry and those who were acutely ill did not participate in our study. 

The questionnaire was implemented electronically in a mobile application. Researchers trained seven questioners to work with this app. They should read questions on mobile screen for patients and insert the patients’ answers to app form. They were also trained to interact with the participants and resolve any possible ambiguities with the questions.

The average time taken to fill out each questionnaire was between 6 to 8 minutes. The number of valid questionnaires collected was 462 from 57 offices and the response rate was 92.4%. The average percentage of missing data was less than 1% per question. Ethics Committee at Mashhad University of Medical Sciences approved all aspects of this study (Reference Number IR.MUMS.fm.REC.1396.253).

The study used a researcher-made survey questionnaire. Critical review of physician and OB/GYN selection literature formed the first draft of the questionnaire. To find relevant papers, we searched the published papers published before 2018 indexed in Pub Med and web of science, using keywords in three groups, including “OB/GYN” and all its mesh terms, words related to “selection” or “choice”, and words related to “preferences,” “satisfaction,” and “priorities”. In addition, we manually searched for the references of relevant articles to identify more publications. Using a Likert scale, we classified the spectrum of answers into five items as follows: highly important, important, does not make a difference, unimportant, and highly unimportant.

Face validity refers to the transparency or relevance of a test as it appears to participants. ^16^ This step was performed both qualitatively and quantitatively. For qualitative assessment of face validity, an expert panel consisted of 10 women expert in survey of the instruments design, nursing, midwifery, obstetrics-gynecology, and medical informatics specialists. These participants considered whether there were any difficulty or potential ambiguities in understanding the words and phrases. Quantitative evaluation of the face validity was performed on 51 remained items via the “item impact score” method and a score ≥1.5 was considered appropriate. ^17^ For this purpose, the questionnaire was distributed among 20 laywomen. Convenience sampling was used to select these laywomen. The inclusion criterion was visiting an OB-GYN specialist at least once.

Content validity was conducted in two stages: qualitative and quantitative. For qualitative content validity, five nursing, midwifery and obstetrics-gynecology specialists were asked to provide corrective views in written form after the careful study of the tool. The importance of grammar compliance, use of appropriate words, importance of questions, placement of questions in their proper place, and the time needed for completion of the designed tool was also emphasized.

For quantitative content validity, content validity ratio (CVR) and content validity index (CVI) were calculated. The necessity of including each item in the questionnaire was estimated using CVR with a minimum threshold of 0.49 based on the Lawshe table. The relevance, simplicity, and clarity of the questionnaire items was estimated using CVI scores with a minimum threshold of 0.79 according to Hyrax. ^18^ Sixteen expert women including medicine, nursing, midwifery, obstetrics-gynecology, social medicine, medical informatics, and health information management specialists participated in this evaluation phase. The numerical means of the panel’s ratings were also calculated, as a method for preventing the loss of items that might be beneficial but did not gain the required CVR score. Cronbach’s alpha was used to measure the instrument reliability. 

Factor analysis (FA) is one of the most practical and useful methods for determining construct validity in methodological research. ^19^ It is considered as a fundamental statistical method due to its power, sensitivity, and ability to adapt according to the research issue. ^20^ FA is conducted in two ways: exploratory factor analysis (EFA) and confirmatory factor analysis (CFA). In this study, both EFA and CFA method was carried out.

EFA was conducted as a pre-test to evaluate the questionnaire items construct validity and dimension reduction. In this phase, a principal component analysis (PCA) was applied to determine the factors influencing OB/GYN selection. In addition, to minimize the issue of cross-loading, orthogonal varimax rotation was used. All items with factor loading >0.40 were retained. 

The number of factors was extracted based on three criteria: (i) the Kaiser criterion (eigenvalues>1); (ii) the inflection point of the scree-plot; and (iii) the interpretability of the factors with an eigenvalues of more than one which were located outside the horizontal line of the scree plot.

CFA method was applied to develop the measurement model of Women’s preferences in selecting an OB/GYN. Measurement model as a base of SEM (Structural Equation Modeling) can assess the simultaneous effect of indicators. According to EFA result, all variables that remained in EFA model were considered in generating CFA model. Several modifications were made due to the fit indices. ^21^ Both EFA and CFA were conducted on the same data (462 record) in this study. Since validity confirmation of EFA-result was not the researchers’ aim, there was no need for the pilot study data and field study data to be different. EFA was performed in SPSS-v23 and CFA in AMOS-v24.

## RESULTS

The number of participants was 462 with a mean age of 30±7.176 years. The minimum age was 18 years and the maximum was 60. Two-thirds of the subjects were housewives and 6 (1.3%) were illiterate. Most of them were married 441 (95.5). Over 370 (80%) of the participants were living in the city, while the others came from neighboring towns and villages. For more than half of the patients, pregnancy and its related care were the reasons for visiting the doctor. Details of the demographic characteristics of the participants are presented in Table 1.

**Table 1:** Sociodemographic characteristics of the participants

Variable	SampleN (%)
Age (years)	
≤20	12 (2.60)
20-30	214 (46.40)
30-40	184 (39.80)
40-50	42 (9.10)
50-60	9 (1.90)
≥ 60	1 (0.20)
Education level	
None	6 (1.30)
Below Diploma	70 (15.20)
Diploma	160 (34.50)
Bachelor’s Degree education	180 (39)
Higher than Bachelor’s Degree	46 (10)
Job status	
Employee	89 (19.30)
Housewife	323 (69.90)
University student	17 (3.70)
Out of Home Part-Time	33 (7.10)
Marital status	
Single	18 (3.90)
Married	441 (95.50)
Other (e.g. widow)	3 (0.60)
Mobile phone	
I have a mobile phone	455 (98.50)
I do not have a mobile phone	7 (1.50)
Pregnancy experience	
I have not been pregnant before	180 (39)
Previous Experience of Pregnancy	282 (61)
Number of OB/GYN visits in the last year	
None	63 (13.60)
1-5	256 (55.50)
6-10	106 (22.90)
≥10	37 (8)
Number of OB/GYN physician visit so far	
I have not visited before	17 (3.70)
Only one gynaecologist	158 (34.20)
Two or more gynaecologists	287 (62.10)
Cause of visit	
Pregnancy Care	240 (52)
Check-up Annually	124 (26.80)
Female Diseases	71 (15.40)
Other	27 (5.80)

### *Item Generation*

**Figure 1:**
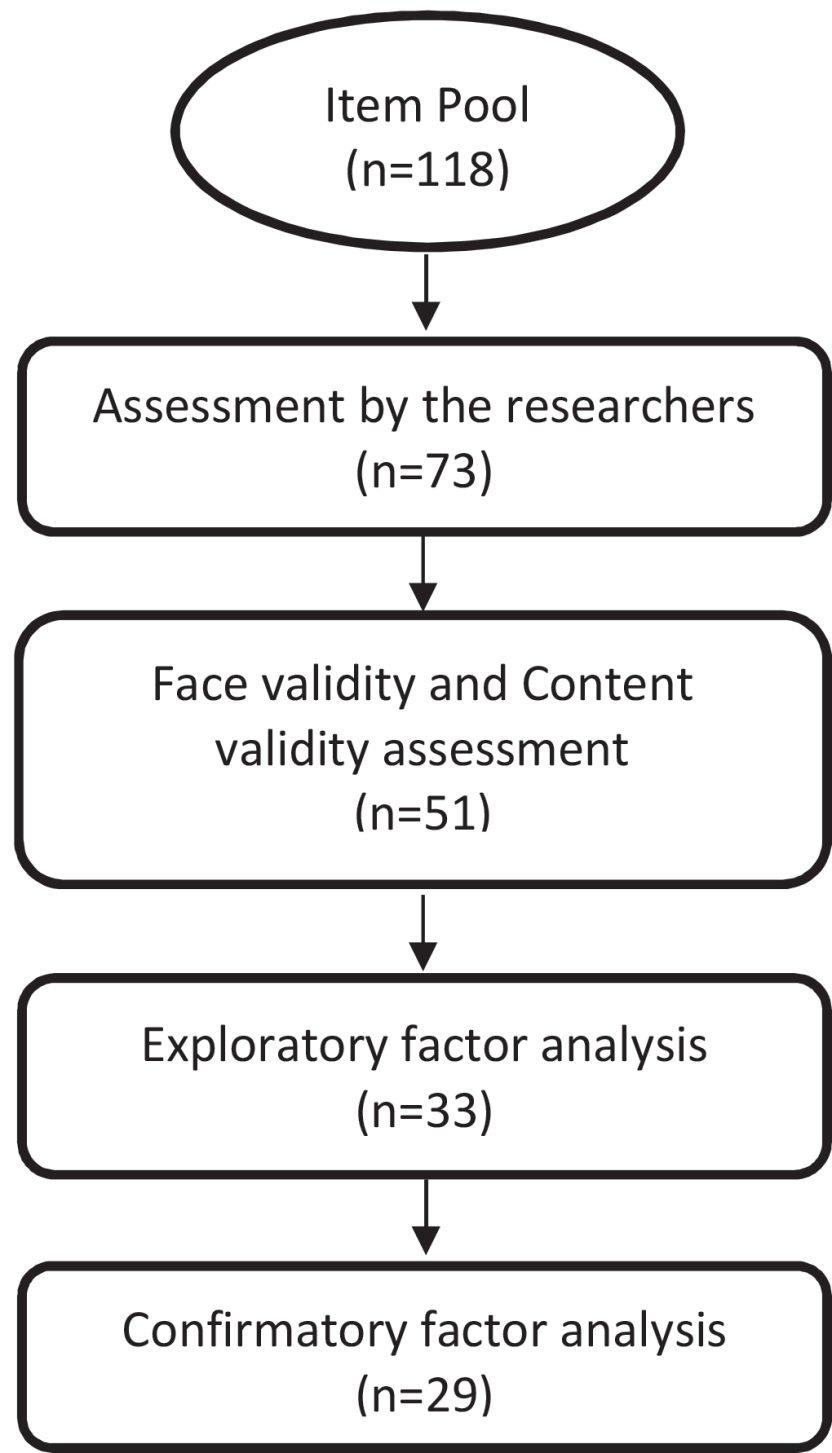
A summary of the instrument development and psychometric evaluation

Figure 1 presents the stages of questionnaire generation and items remained in each stage. The item pool originally contained 118 items. The researchers reviewed item overlapping to delete or merge. The primary questionnaire with 73 items was given to the expert panel to examine the qualitative face validity. In this step, 22 items were merged or deleted from the item pool. Item impact scores as quantitative face validity are shown in Table 2. 

**Table 2:** Preliminary Questionnaire items and results of the content validity

	CVR	CVI Relevancy	Item Impact Score
1. Physician’s individual and professional characteristics			
Reputation	0.63	0.69	2.75
Experience in role	0.80	0.68	3.48
Physician age	0.60	0.81	1.22
Physician gender (patient examination)	1.00	0.87	2.91
Physician gender (surgery/delivery)	1.00	0.87	2.92
University affiliation	0.60	0.56	0.78
Fellowships	0.45*	-	3.35
Department location (hospital, clinic, office)	0.63	0.43	1.85
Hospital affiliation (public or private)	0.63	0.43	2.18
Special practices offered by the physician	0.82		3.39
2. Hospital condition in surgery/delivery			
Attitude to cesarean or normal delivery	1.00	0.93	2.85
Obstetrician’s presence at the patient’s bedside in delivery	1.00	1.00	3.64
Hospital affiliation (surgery/delivery)	0.82	0.75	3.27
3. Behavioral and communicational skills			
Paying attention to the patient	1	0.93	4.23
Respecting the patient during the examination	1	0.81	4.17
Verbal communication skills	1	0.81	3.98
Honesty	1	0.81	4.11
Confidentiality	1	0.93	4.06
Private consultation	1	0.93	3.74
Private examination	1	0.93	4.02
Flexibility and collaborating with patient	1	0.81	3.54
Training (appropriate solutions)	1	1	4.12
4. Physician accessibility and availability			
Proximity of the office to home	0.60	0.93	1.32
Convenience access to the office	0.82	0.87	2.12
Variety of appointment procedures	0.64	0.68	2.67
Ease of making appointments	1	0.87	3.43
Information about office hours	0.82	0.87	3.63
Information about hours of other hospitals/clinics	0.64	0.75	2.92
Getting an appointment as soon as possible	0.46*	-	2.39
Out of hours availability	0.46*	-	3.76
Possibility of asking questions via social media	0.45*	-	3.91
5. Physical and environmental variables of the office			
Physical office facilities	0.82	0.75	2.99
Accompaniment by spouse or friends	0.45*	-	1.81
Waiting room capacity	0.82	0.87	2.71
Office amenities	0.45*	-	2.50
Having spouse’s waiting room	0.27*	-	2.61
Having lactating room	0.27*	-	2.61
Office entertainment facilities	0.27*	-	1.56
Waiting times	1	0.81	3.97
Staff behavior	0.82	0.87	4.10
6. Cost and insurance			
Cost of practices	0.64	0.75	3.08
Surgery/delivery cost	0.82	0.81	3.44
Having insurance coverage	1	0.87	3.85
Supplementary insurance	0.64	0.62	3.60
7. Special services			
Office diagnostic equipment	0.82	0.75	3.32
Proximity of the office to para-clinical services	0.45*	-	3.11
Providing information about para-clinical services	1	0.68	2.94
8. Advertising and recommendations			
Friends and family recommendation	0.82	1.91	2.88
Other doctors/colleagues recommendation	0.64	1.73	3.10
Former patients satisfaction	0.45	1.7	3.59
Activity in social media	0.4	1.56	1.14

* CVI did not calculated because CVR<0.49. This item was candidate of deletion

Table 2 also demonstrates the CVR, CVI results. S-CVI turned out to be 0.80. According to the CVI/CVR, the item impact scores, and the numerical means of the panel’s ratings, 10 items were candidate for exclusion from the questionnaire. Fung quotes Rogers that more ambiguous items should be seen “as yellow flashing lights” and should be treated and eliminated with caution. ^22^ Therefore, the research team decided to keep the above items with minor changes.

### *Findings of the EFA*

**Figure 2:**
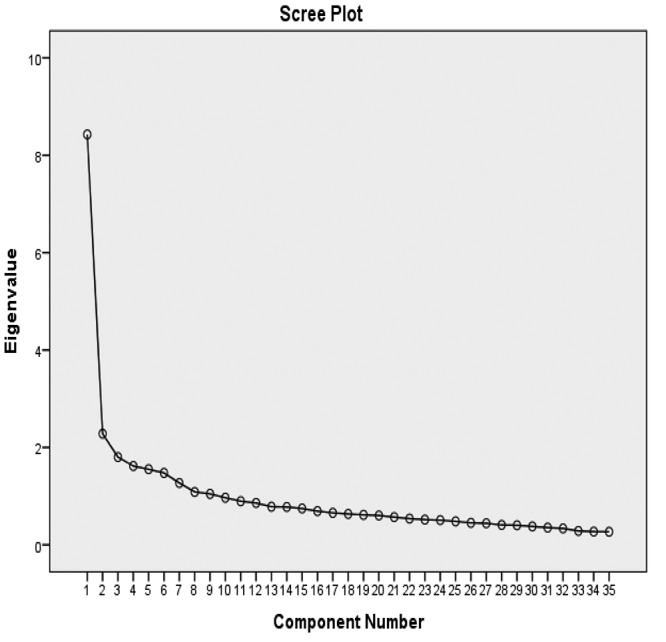
Scree plot

All of 51 questionnaire items were used in the EFA to avoid loss of data. The final EFA model with 33 items was approved. The final value of the Kaiser-Meyer-Olkin (KMO) test was 0.88 (P<0.001), so the sampling adequacy was confirmed. Bartlett’s test of sphericity was significant (χ2=4640.070, df=406, P<0.001), which indicates sufficient correlation among the items in the data matrix. Six factors were extracted based on eigenvalues and inflection point of the scree-plot. Cumulative variance explained by these six factors was 52.657% (Figure 2).

Items, which did not seem to belong to EFA model, were removed. Finally, seventeen items were deleted due to a factor loading below 0.4 and cross loading. These items were physician’s gender, age, university, and department as well as attitude to cesarean/normal delivery, presence at the patient’s bedside in delivery time, flexibility and collaboration with patients, office hour’s information, possibility of asking questions via social media, and activity in social media. Table 3 shows the factor loading of each item after varimax rotating.

**Table 3:** Factor loadings after varimax rotating for women preferences in obstetrician and gynecologist selection

Items	Factor 1	Factor 2	Factor 3	Factor 4	Factor 5	Factor 6
Providing a safe and private place during examination	0.77					
Secrecy and confidentiality of examination	0.71					
Honesty	0.66					
Providing a safe and private place during consultation	0.66					
Paying attention to the patient and listening to them eagerly	0.64					
Giving appropriate solutions to patient problems (training)	0.63					
Verbal communicational skill	0.59					
Staff behavior	0.56					
Access to the physician out of hour in emergency cases	0.49					
Waiting times	0.43					
Easy access to information about physician office hours		0.71				
Making appointments easily		0.65				
Getting an appointment as soon as possible		0.59				
Variety of appointment procedures such as telephone, website and walk-in		0.56				
Convenience access to the office for example traffic, parking options, and public transportation availability		0.53				
Proximity of office to home		0.45				
Waiting room capacity and welcoming			0.63			
Having lactating room			0.60			
Office amenities such as elevator, restrooms, TV and frig			0.60			
Having spouse’s waiting room			0.54			
Office condition and facilities for instance cleanliness and proper ventilating			0.53			
Office diagnostic equipment such as ultrasound and sonography devices				0.74		
Special practices offered by OB-GYN (such as ultrasound, laparoscopy, pap smear test and hysteroscopy; fellowships)				0.62		
Proximity of the office to para-clinical services				0.60		
Accessibility of information about para-clinical services				0.59		
Having insurance coverage					0.83	
Support supplementary insurance					0.83	
Surgery and delivery cost					0.63	
Experience in role						0.62
Reputation						0.60
Recommendation from friends and family						0.56
Former patient satisfaction						0.55
Recommendation from other doctors/colleagues						0.44

Construct reliability test demonstrate the internal consistency of the whole questionnaire (Cronbach’s alpha=0.88). The items inside each factor also have a relatively high internal consistency (Table 4). 

**Table 4:** Reliability of the factors

Number of factor	Factor	Number of Item	Cronbach’s α (N=462)	ICC (95% CI) (N=462)
1	Behavioral and communication skills	10	0.86	0.86 (0.85-0.88)
2	Accessibility and availability	6	0.69	0.69 (0.65-0.73)
3	Office environment	5	0.71	0.71 (0.67-0.75)
4	Special procedures and para-clinical service	4	0.67	0.67 (0.62-0.72)
5	Cost and insurance	3	0.75	0.75 (0.70-0.78)
6	Professionalism and recommendation	5	0.60	0.60 (0.54-0.65)
	Total	33	0.88	0.88 (0.86-0.89)

### *Findings of the CFA: Measurement Model*

**Figure 3:**
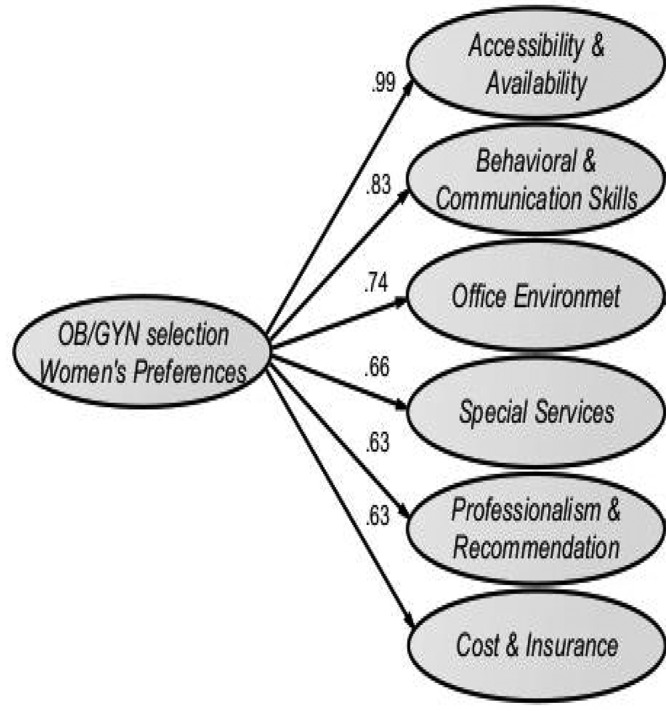
Latent variables of final measurement model of women’s preferences when selecting an OB/GYN in the private sector. Arc numbers are factor loading of latent variables.

Final measurement model of women’s preferences in selecting an OB/GYN suggests 6 latent variables and 29 observed variables based on hypothesized model obtained from EFA and new modifications in CFA. Figure 3 shows the latent variables and their factor loadings. As shown, accessibility and availability factor had the most factor loading (0.99) professionalism & recommendation, and cost and insurance got the least factor loading (0.63). The research team added OB-GYN gender dimension in the final measurement model and assessed its role in patient preferences again. Since factor loading of this added dimension was 0.13, which was lower than the threshold (0.4), they removed it from the final measurement model. 

**Figure 4:**
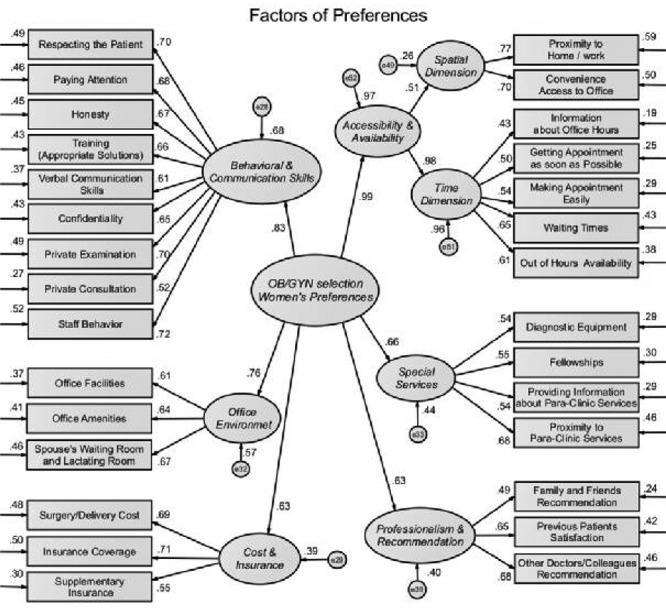
Final measurement model of women’s preferences when selecting an OB/GYN in the private sector. It is the direct output from AMOS. Arc labels are factor loading of questionnaire items as observed variables.

Figure 4 indicates latent variables and their related observational variables of the final measurement model. As shown, “Accessibility and availability” factor is divided into time and spatial dimensions. Time dimension is related to rapid access to services and spatial dimension refers to proximity and convenient access, for example traffic, parking options, and public transportation availability. Other modifications in CFA phase were elimination of “office welcoming environment” and “waiting room capacity” in office environment dimension, “Experience in Role” and “physician Reputation” in professionalism dimension. These items got factor loading lower than the threshold (0.4). 

### *Evaluation of Measurement Model: Model Fit Indicators*

As shown in Figure 4, most items had strong factor loadings (above 0.5) with their corresponding latent constructs. The correlation between each factor and its underlying items has a t-value higher than 1.96, indicating that the relationships are significant. The values of the fit indices for each factor are shown in Table 5, which are within or almost within acceptable ranges for all indices, so that the efficiency of our measurement model is verified.

**Table 5:** Values of fit indices for final measurement model

Index	Title	Desired Limit	Reported Value
CMIN/DF	Normed chi-square (c^2^/df)	Under 3	1.61
RMR	Root mean residual	Near zero	0.03
GFI	Goodness of fit index	0.90	0.95
AGFI	Adjusted goodness of fit index	0.90	0.90
NFI	Normed fit index	0.90	0.88
IFI	Incremental fit Index	0.90	0.95
CFI	Comparison fit index	0.90	0.95
RMSEA	Root mean square error of approximation	Under 0.08	0.03

## DISCUSSION

The purpose of this study was to investigate, construct, and validate a measurement model of women’s preferences when selecting an OB/GYN in the private sector based on a valid and reliable instrument. We introduce a measurement model using a comprehensive list of items and factors. In the process of developing the model, several fit indices were measured. 

### *Accessibility and Availability*

Accessibility and availability are considered as one of the infra-structural aspects of treatment quality based on the convenience of receiving health services. This indicator is measured based on three factors: accessibility, availability, affordability. In contrast to most studies, in our final model this factor obtained the highest score of all the factors. The time dimension of the availability such as waiting time, out of hour availability in pregnancy conditions and getting appointment as soon as possible are more important than the spatial dimension. This finding supports the previous studies although none of them found this factor as the most important factor. ^6 , 7 , 23 - 25^ The absence of diversity in the turning systems, low presence of experts in this field in online turning systems, and uncommon turning in some of the offices that leads to long queues are some of the reasons for the fact that availability was very important for our participants. 

Due to the congestion of specialist doctor’s office in one of the urban areas, as well as traffic jams in this area, some factors such as proximity and convenience to get to the office as accessibility factor were important in our model. In such a situation, proposing a way to provide accurate, real-time information about the presence of specialists in their offices could help to reduce the patients’ confusion and waste of their time. 

### *Behavioral and Communicational Skills*

The second most important factor in our model is the behavioral and communicational skills of the physicians and their staff. This finding is consistent with previous studies. ^8 , 23 , 26^ Doctors’ communicational skill is the cornerstone of medical practice, and the patients assess the physicians’ expertise by their behavioral and communicational skills. ^24 , 27^ Hamelin refers to the non-verbal interpersonal skills of doctor as the “Art of Care” and describes it as “the communication of caring, concern, sincerity, compassion, and respect”. ^28^ The hospitality and respectful behavior of the office staff and assistants also lead to the patients’ satisfaction. ^7 , 25 , 29^ Thus, physicians should pay much attention to the way that their staff deal with patients.

The importance placed by the participants on doctor’s sensitivity toward patients in privacy leads to the appearance of a confidentiality item in the model. Similar studies have also emphasized the patient privacy. ^30 , 31^ For example, a study considered a number of items, including: practices surrounding the privacy of the patient when changing clothes, patient examination taking place behind a curtain or in another room, a nurse accompanying the patient during the examination, reducing the amount of conversation during examination except for technical cases, and minimizing the amount of eye contact during examination. ^10^

### *Environmental Conditions of the Office*

The physician’s office environment is an influential factor in our model. In a study in the United States (2005), physicians were advised to optimize the quality of the office environment to make it attractive to the patients. ^32^ Since patients spend a noticeable waiting time in OB/GYN’s offices, facilities such as restrooms, lactating room, and elevator are remarkably significant. Office amenities such as convenient parking, exam room design, appropriate sofa, and entertainment tools also affect the patients’ satisfaction and preferences. 

### *Special Procedures and Services*

There are various special practices carried out by an OB/GYN. Experts in this field accept patients according to their specializations such as oncology, perinatology and laparoscopy. Therefore, providing accurate information about which special practices can be performed at an OB/GYN office leads to a reduction in unrelated patient referrals. Providing information about paraclinical services, such as laboratory services, sonography, mammography, and laparoscopy, is another concern of women visiting an OB/GYN. 

### *Recommendation and Professionalism*

Word of mouth, i.e. recommendations from family, friends, and coworkers, as well as previous patient satisfaction, influence the women’s decision-making in OB/GYN selection. However, the model showed that coworkers’ recommendation is more impressive. These findings are consistent with those of similar studies. ^8 , 33^ Although qualitative criteria such as expertise, treatment outcome, and physician diagnostic accuracy have been considered as “professionalism” by several texts, patients are not usually in a position to evaluate the quality of the doctor and his/her expertise; instead, they consider recommendations by other patients. ^34^ Elimination of reputation in final model supports the idea of covariate reputation and recommendation. In addition, it seems that OB-GYN’s work experience is not a priority for patients. 

### *Cost and Insurance*

According to the model, insurance coverage and delivery cost are the least important variables. This finding confirms those of similar studies. ^35 - 37^ There are two main insurance types in Iran. Basic health insurance organizations cover a significant portion of medical costs. Supplementary health insurances are also provided voluntarily to cover the patients’ medical costs that the basic insurance types do not support. Delivery, cancer, surgical practices and diagnostic test such as sonography cost in OB/GYN field are some of this coverage. Patients may decide to change OB/GYN based on their insurance coverage. In some cases, the gynecologist’s hospital affiliation (governmental or private) and type of delivery (vaginal or cesarean) are important issues in selecting gynecologist/obstetrician according to the insurance support.

Physician’s gender is not included in the model statistically. It implies that under the simultaneous effect of other factors, gender preference loses its key role for patients of OB-GYN’s offices. This finding supports some studies ^6 , 9^ although it differs from other earlier ones. ^10 , 13^

In order to reduce the sampling bias, we decided to recruit all eligible women during a specific period. An attempt was made to collect a representative sample from an economic, social, and cultural perspective. We used an interview-based mobile application instrument rather than self-completion in order to increase the response rate through direct personal contact and interaction with participants. Since the mobile application was used to enter the patient’s answers, the process of data gathering led to easy and accurate analysis. In order to decrease the response bias, interviewers approached women as an independent researcher.

The limitation of this study is that it did not consider the quality parameters such as treatment outcome and physicians’ diagnosis accuracy in the model, due to the lack of efficient tools for measuring these parameters. Lack of information to assess the characteristics of non-respondents can be considered as another limitation. This could cause bias in the results because motivated participants may differ from non-respondents. Although the sampling was carried out in a city level, we still think our model will be applied for low-income and middle-income countries, especially in the Middle-East region with the same culture as Iran.

## CONCLUSION

The present study is a comprehensive examination of all the hidden non-clinical aspects of choosing an OB/GYN in the private sector. To achieve this goal, a valid and reliable instrument was developed. EFA and CFA were applied to validate the construct and create measurement model, respectively. Although the relationship between a health professional and a patient is extremely complex, our final measurement model efficiently represents the simultaneous effect of women’s preferences in OB/GYN selection 

Physician availability and office accessibility, physician and staff behavioral and communicational skills, recommendation by other colleagues and patients as a qualified specialist, and providing special services and cost & insurance components are in the final model. Improving the office environment and providing accurate information about paraclinical services can increase the patients’ satisfaction and relaxation. In our study, when OB/GYN gender was considered alongside other factors simultaneously, the results indicated that the gender of the OB/GYN is not the patient’s priority.

The model of our study could be used to develop a recommendation system, which suggests suitable OB/GYNs according to the patients’ needs and preferences. It will be helpful for policymakers to improve the women’s access to these critical health services and their satisfaction. On the other hand, obstetrics and gynecology specialists can use our model to efficiently improve their service delivery by recognizing the impact of each factor from the patient’s point of view. We believe that our research method will serve as a base for future studies on other medical practices, without a significant degradation in performance.

## References

[1] Van de Walle S, Marien S (2017). Choice in public services: A multilevel analysis of doctor choice in 22 countries. Administration & Society.

[2] Kim K, Ahn S, Lee B ( 2018). Factors associated with patients’ choice of physician in the Korean population: Database analyses of a tertiary hospital. PloS One.

[3] Murray CJL, Evans DB (2003). Health systems performance assessment: debates, methods and empiricism.

[4] Janssen SM, Lagro-Janssen AL ( 2012). Physician’s gender, communication style, patient preferences and patient satisfaction in gynecology and obstetrics: a systematic review. Patient Education and Counseling.

[5] Vos HMM (2014). Risk factors in women’s health in different stages of life. [thesis].

[6] Amir H, Hazan M, Hasson J ( 2012). Gender preference of obstetricians and gynecologists by ultra-orthodox Jewish women. Open Access Scientific Reports.

[7] Amir H, Gophen R, Amir Levy Y (2015). Obstetricians nd gynecologists: which characteristics do Israeli lesbians prefer?. The Journal of Obstetrics and Gynaecology Research.

[8] Amer-Alshiek, Alshiek T, Amir Levy YJ ( 2015). Israeli Druze women’s sex preferences when choosing obstetricians and gynecologists. Israel Journal of Health Policy Research.

[9] Amir H, Abokaf H, Levy YA ( 2018). Bedouin Women’s Gender Preferences When Choosing Obstetricians and Gynecologists. Journal of Immigrant and Minority Health.

[10] Willis E, King D, Dwyer J ( 2017). Women and Gynaecological Cancer: Gender and the Doctor–Patient Relationship. Topoi.

[11] Makam A, Mallappa Saroja CS, Edwards G (2010). Do women seeking care from obstetrician-gynaecologists prefer to see a female or a male doctor?. Archives of Gynecology and Obstetrics.

[12] Balayla J ( 2011). Male physicians treating female patients: issues, controversies and gynecology. McGill Journal of Medicine.

[13] Schnatz PF, Murphy JL, O’Sullivan DM, Sorosky JI ( 2007). Patient choice: comparing criteria for selecting an obstetrician-gynecologist based on image, gender, and professional attributes. American Journal of Obstetrics & Gynecology.

[14] McLean M, Al Yahyaei F, Al Mansoori M ( 2012). Muslim women’s physician preference: beyond obstetrics and gynecology. Health Care for Women International.

[15] Amouzagar S, Mojaradi Z, Izanloo A ( 2016). Qualitative examination of health tourism and its challenges. International Journal of Travel Medicine and Global Health.

[16] Gravetter FJ, Forzano LAB (2017). Research methods for the behavioral sciences.

[17] Sharif Nia H, Pahlevan Sharif S, Lehto RH ( 2017). Development and psychometric evaluation of a Persian version of the Death Depression Scale-Revised: a cross-cultural adaptation for patients with advanced cancer. Japanese Journal of Clinical Oncology.

[18] Polit DF, Beck CT, Owen SV ( 2007). Is the CVI an acceptable indicator of content validity? Appraisal and recommendations. Research in Nursing & Health.

[19] Waltz CF, Strickland O, Lenz ER (2010). Measurement in nursing and health research.

[20] Kerlinger FN (1986). Foundations of behavioral research.

[21] Mammen PM, Asokan MK, Russell S ( 2018). The confirmatory factor analysis of the original brief intellectual disability scale and alternative models. Indian Journal of Psychological Medicine.

[22] Fung K (2011). The initial development and content validity of an Asperger’s Syndrome self-screening instrument for adults. [thesis].

[23] Al-Briek A, Al-Barrak A, Al-Johi K ( 2018). Factors that Influence Patients in Choosing Their Treating Physicians in the Private Sector in Saudi Arabia. American Journal of Public Health Research.

[24] Perrault EK, Smreker KC ( 2013). What can we learn from physicians’ online biographies to help in choosing a doctor? Not much. A content analysis of primary care physician biographies. Journal of Communication in Healthcare.

[25] Rogo-Gupta LJ, Haunschild C, Altamirano J ( 2018). Physician Gender Is Associated with Press Ganey Patient Satisfaction Scores in Outpatient Gynecology. Women’s Health Issues.

[26] Bal MD, Yılmaz SD, Beji NZ, Uludağ S ( 2014). Muslim women choice for gender of obstetricians and gynecologist in Turkey. Journal of Human Sciences.

[27] Kashgary A, Alsolaimani R, Mosli M, Faraj S ( 2017). The role of mobile devices in doctor-patient communication: A systematic review and meta-analysis. Journal of Telemedicine and Telecare.

[28] Hamelin ND, Nikolis A, Armano J ( 2012). Evaluation of factors influencing confidence and trust in the patient-physician relationship: a survey of patient in a hand clinic. Chirurgie de la Main.

[29] Lee Y ( 2018). Patients’ perception and adherence to vaginal dilator therapy: a systematic review and synthesis employing symbolic interactionism. Patient Preference and Adherence.

[30] Shahawy S, Deshpande NA, Nour NM ( 2015). Cross-cultural obstetric and gynecologic care of Muslim patients. Obstetrics & Gynecology.

[31] Magnezi R, Bergman LC, Urowitz S ( 2015). Would your patient prefer to be considered your friend? patient preferences in physician relationships. Health Education & Behavior.

[32] Johnson AM, Schnatz PF, Kelsey AM, Ohannessian CM ( 2005). Do women prefer care from female or male obstetrician-gynecologists? A study of patient gender preference. The Journal of the American Osteopathic Association.

[33] Amir H, Tibi Y, Groutz A ( 2012). Unpredicted gender preference of obstetricians and gynecologists by Muslim Israeli-Arab women. Patient Education and Counseling.

[34] Cooley DO, Madupu V ( 2009). How did you find your physician? An exploratory investigation into the types of information sources used to select physicians. International Journal of Pharmaceutical and Healthcare Marketing.

[35] Nan Liu, Finkelstein SR, Kruk ME, Rosenthal D ( 2018). When Waiting to See a Doctor Is Less Irritating: Understanding Patient Preferences and Choice Behavior in Appointment Scheduling. Management Science.

[36] Victoor A, Delnoij DM, Friele RD, Rademakers JJ ( 2012). Determinants of patient choice of healthcare providers: a scoping review. BMC Health Services Research.

[37] Yahanda AT, Lafaro KJ, Spolverato G, Pawlik TM ( 2016). A systematic review of the factors that patients use to choose their surgeon. World Journal of Surgery.

